# Group psychological intervention for people affected by conflict in Central African republic

**DOI:** 10.1192/j.eurpsy.2021.304

**Published:** 2021-08-13

**Authors:** E. Dozio, C. Bizouerne

**Affiliations:** 1 Mental Health And Care Practices, Action Contre la Faim, Paris, France; 2 Mental Health And Care Practices, Gender And Protection, Action contre la Faim, Paris, France

**Keywords:** PM+, Central African Republic, Cultural Psychology, social cohesion

## Abstract

**Introduction:**

A large part of the Central African population has been exposed to potentially traumatic events as a result of the recent conflict, which has led to the breakdown of social ties.

**Objectives:**

Faced with this situation, the NGO Action contre la Faim proposed a psychosocial intervention aimed at helping the displaced people to reduce their psychological suffering and strengthen individual and community resilience.

**Methods:**

After psychoeducation sessions organized in communities affected by the conflict, people identified with traumatic symptoms are invited to participate in a psychological support intervention. The protocol used is based on the Problem Management Plus (PM+), developed by the WHO. The approach was adapted in groups to reach more suffering people and also to take advantage of the group dynamic in the possibility of recovering and developing better resilience.

**Results:**

946 IDPs in the country’s capital, participated in the group intervention led by a team of paraprofessionals. Data collected from 111 participants show that after five weeks of intervention, there was a significant reduction in post-traumatic symptoms (PCL-5) and functional impairment (WHODAS). These results were confirmed during the post-intervention evaluation four weeks later. In addiction, participants declared that they had observed effects on their ability to live together in the community and to regain social cohesion.

**Conclusions:**

This experience gives encouraging results with regard to the feasibility and replicability of the group protocol, taking into account specific cultural and contextual adaptations.

**Disclosure:**

No significant relationships.

